# Building an economy for health for all: a call for papers

**DOI:** 10.2471/BLT.23.290078

**Published:** 2023-05-01

**Authors:** Ritu Sadana, Rajat Khosla, Rachel Gisselquist, Kunal Sen

**Affiliations:** a World Health Organization Secretariat, Council on the Economics of Health for All, Avenue Appia 20, 1211 Geneva 27, Switzerland.; b United Nations University International Institute for Global Health, Kuala Lumpur, Malaysia.; c United Nations University World Institute for Development Economics Research, Helsinki, Finland.

The financial and economic choices societies make determine whether all individuals can enjoy the right to health. Crises quickly expose the values and interests that drive decisions towards health, as shown by the coronavirus disease 2019 (COVID-19) pandemic. The unequal impact of the pandemic exposed existing structural inequalities and economic relationships, including the already negative impact of loan and austerity conditionalities. For example, the average government expenditure on health across low-income countries, as a percentage of gross domestic product, was lower just before the pandemic in 2019 (1.3%) than it was in 2000 (1.4%).^1^

In response, the World Health Organization (WHO) Council on the Economics of Health for All, chaired by Mariana Mazzucato, calls for a radical reorientation of economies for health, replacing the prevailing paradigm that good health is only an instrument for economic growth.^2^ This new narrative promotes a vision that every person can flourish physically, mentally and emotionally, and that all people are endowed with the capabilities to lead a life of dignity, opportunity and community, as part of a healthy living planet.^3^ Reorienting economies to deliver health for all requires collective multisectoral actions. The council’s work focuses on four themes to build economies for health: (i) clarifying values and how progress is measured;^3^ (ii) financing health as a long-term investment;^4^ (iii) governing health innovation through strategies engaging public and private sectors to collaborate;^5^ and (iv) ensuring strong public sector capacity that drives policies and creates an enabling environment towards the common good.^6^

Yet in many countries private and commercial interests drive national economic and financial policies and legislation. For example, pooled financing for universal health coverage (UHC) remains widely contested, despite ample evidence that it is the preferred approach to provide effective services to all without financial burden.^7^^,^^8^ Moreover, the council documents that many international and national policies have failed to govern health innovation.^5^ WHO data reveal that many countries are unable to finance basic primary health-care services that reach all their populations, and some high-income countries are struggling to maintain existing services without increasing financial burden on individuals and households.^9^

The climate crisis and relationship to health adds to the importance of rethinking what societies’ goals should be, and reshaping international, regional and domestic macroeconomic and fiscal policies to reach these goals. A transition to a circular economy that respects planetary boundaries is needed. Such transition also requires regenerative business models, including in the health sector, such as reducing emissions in facilities and not overproducing products that are inappropriately used, such as antibiotics.

The council advocates using a full range of policy levers to redress the power asymmetries and entrenched economic interests that have hampered progress towards financing health for all people. The council’s final report, to be released in May 2023, will call for adequate fiscal space for UHC and broader determinants of health. For example, policies and regulations can put in place progressive and fair taxation, whether imposed on corporations,^10^ individuals, or on health-damaging products that contribute to the increasing burden of noncommunicable diseases.^11^ Commercial imperatives, charitable efforts, and reliance on women’s unpaid work are insufficient to deliver needed health products to all people on time, as the limited distribution of COVID-19 vaccines to low-income countries revealed.

The council advocates moving away from solutions that attempt to fix market failures, as these reinforce the problems of the existing ecosystem, and to develop instead mission- and outcome-oriented models collectively governed in the public interest.^12^ Overall, new economic policies and business strategies have the potential to influence and accelerate the realization of the right to health.

*The Bulletin of the World Health Organization* will publish a theme issue on global and national approaches to reorient economies towards health, using different policy and programmatic levers and methods in varied settings. Submitted articles should be inspired by the four themes and forthcoming recommendations of the WHO Council on the Economics of Health for All, particularly how societies should think about purpose, value and economic activity that can inform a new social contract between governments and people that connects the health of people and health of the planet for sustainable development. We welcome papers that expand our current knowledge by presenting economic policies relevant to increasing the level of financing for health from a whole-of-government perspective, and the quality of finance that shapes alliances across public, private, civil society, international and domestic actors.

The deadline for submission is 31 August 2023. Manuscripts should be submitted in accordance with the *Bulletin’s *guidelines for contributors (available at: https://www.who.int/publications/journals/bulletin/contributors/guidelines-for-contributors) and the cover letter should mention this call for papers.
